# Exploring the addictive processes in repetitive self-harm through a grounded theory approach: *“It always felt like a choice until it didn’t”*

**DOI:** 10.1371/journal.pone.0328914

**Published:** 2025-08-14

**Authors:** Millie Witcher, Sarah Rowe, Sally Marlow, Jennifer Heath

**Affiliations:** 1 Doctorate Programme in Clinical Psychology, School of Health, Medicine and Life Sciences, University of Hertfordshire, Hatfield, Hertfordshire, United Kingdom; 2 Mental Health Sciences Programme, Division of Psychiatry, University College London, London, United Kingdom; 3 Institute of Psychiatry, Psychology and Neuroscience, Kings College London, London, United Kingdom; Chongqing Normal University, CHINA

## Abstract

**Introduction:**

There is limited empirical research to suggest repetitive self-harm can be understood or conceptualised as an addictive behaviour. However, few empirical studies have investigated this and yielded conflicting results. This study aims to explore to what extent can repetitive self-harm be conceptualized as an addictive behaviour.

**Methods:**

The study employed the principles of Constructivist Grounded Theory to guide the collection and analysis of data from 15 adults with current or past experience of repetitive self-harm.

**Results:**

Thirteen categories were identified within the data that depicted participants journeys with self-harm over time. ‘Starting’ to self-harm (category 1) and participants’ description of ‘needing to punish myself’ (category 2) led to self-harm ‘feeling addictive’ (category 3). Once self-harm had become repetitive, ‘having the urge to self-harm’ (category 4) and experiencing a ‘conflicting relationship with self-harm and self’ was ongoing for participants (category 5). Throughout each incidence of self-harm, participants described a “cycle of self-harm”, in which self-harm had different functions and consequences: ‘managing emotions’ (category 6), ‘allowing me to function’ (category 7), ‘caring for myself’ (category 8), ‘controlling’ (category 9) and ‘feeling guilt and shame after self-harm’ (category 10). All participants described ‘responding to other’s reactions’ to their self-harm (category 11), six discussed ‘breaking the self-harm cycle’ (category 12) and six participants described ‘relapsing’ (category 13) and returning to self-harm following a period of abstinence.

**Conclusion:**

This study has provided a conceptual model of processes that maintain engagement in repetitive self-harm, discussed in relation to addiction literature. Clinical practice could consider working alongside the client to identify where they feel they are within the self-harm cycle in relation to changing their self-harm behaviours.

## Introduction

National Institute for Health and Care Excellence (NICE) guidelines define self-harm as “intentional self-poisoning or injury, irrespective of the apparent purpose of the act” and as “an expression of personal distress, not an illness” [1]. Intentional self-harm is the term used within the eleventh version of the International Classification of Diseases [2], World Health Organisation (WHO), and the diagnostic criteria employed within the National Health Service (NHS). For the purpose of this research, the term self-harm will be used to describe: intentional cutting, burning, branding, scratching, picking at skin or reopening wounds, biting, head banging, hair pulling, hitting, and bone breaking. The term self-harm is used as it does not imply motive or intent of the behaviour. Self-harm will not be used within this study to refer to harm arising from: overeating, body piercing, body tattooing, excessive consumption of alcohol or recreational drugs, restricted eating or starvation arising from eating difficulties or accidental harm to oneself.

The rationale for not including harm arising from the above was because this aligns with the current NICE guidelines’ definition [1] and limited definitions found within existing literature. Self-harm can be either episodic or repetitive [3], with the former occurring more during adolescence [4] and the latter occurring as an individual gets older. Findings from non-clinical populations postulate a cut off of five or more occurrences of self-harm within a 12-month period as the distinction for repetitive self-harm [1,4]. Repetitive self-harm is common, with approximately 20% of individuals repeating self-harm within one year [6].

Research into the epidemiology of self-harm has largely occurred within secondary healthcare settings [7], predominantly inpatient settings [8], with adolescent samples [9]. Previous research estimated 220,000 presentations to Accident and Emergency departments occur annually in England as a result of self-harm [8]. An epidemiological study within primary care settings between 2001−2013 found rates of self-harm to be rising [7], with significantly higher rates of self-harm amongst women and younger age groups. However, incident rates of self-harm are unlikely to include the many individuals who self-harm and do not seek medical intervention. The Global Burden of Disease study estimated 14.6 million people are affected yearly by self-harm and an analysis of self-harm presentations to hospitals in England [10] estimated 228,075 presentations. Similarly, a retrospective analysis of general hospitals costs estimated the cost to the NHS to be £162 million annually [11]. During the COVID-19 pandemic, evidence from 62 emergency departments across 25 countries (including the UK) found rates of self-harm doubled during March and April 2020 and 2021 [12], suggesting current incidence rates and cost to the NHS are higher than previously measured.

The accepted definition of addiction includes, to some extent, a state of physiological adaptation and subsequent dependence on a drug within the body. However, researchers believe it is important to distinguish between ‘physical dependence’ and addiction. Physical dependence is defined as a state of physiological adaption to a substance that must be taken in order to prevent withdrawal effects [13]. The DSM-IV replaced the term ‘dependence’ with ‘addiction’ in 2013. Addictive behaviours are thought to differ from ‘physical dependence’ and addiction as they have not yet been found to have a physiological response or dependence. There is a body of evidence positing a variety of behaviours that can be considered addictive [10]. Marlatt et al. [14] define addictive behaviour as “a repetitive habit pattern which increases the risk of associated personal and social problems”, associated with a loss of control. Many addictive behaviours are not included in either the DSM-5 [15] or ICD-11 [2] at present, mainly due to insufficient evidence on whether these repetitive behaviours can be characterized as a form of addiction [16].

Repetitive self-harm may have addictive qualities [17]; however, it is not currently identified as an addictive behaviour. Faye [18] presents a theoretical rationale for the conceptualisation of repetitive self-harm as an addictive behaviour, drawing similarities between aversive withdrawal effects experienced by drug users and an increase in negative emotions prior to self-harm. Self-harm literature has highlighted tension-releasing effects [12], which can be reinforcing through repeated use. Nixon et al. [4] utilised a self-report measure of addictive aspects of non-suicidal self-injury (NSSI), adapted from the DSM-IV substance dependence criteria, and found the “urge” to self-harm to be daily within almost 80% of their sample of hospitalised adolescents. Blasco-Fontecilla et al. 19) theorised both NSSI and suicidal behaviour can be conceptualised as addictions and advocate for the use of an addictive model of self-harm. Research also evidences activation of the opioid and dopaminergic systems through self-harm that provide brief relief from emotional and psychological pain, acknowledging the overlap between this and other addictive behaviours [20,21].

Current literature also offers alternative models of self-harm including affect-regulation, self-punishment [22], and anti-dissociation [23,24]. Research posits individuals who engage in repetitive self-harm may produce less endogenous opioids and a function of this behaviour may be to simulate the endogenous opioid system, producing relieving or relaxing effects [25,26]. The opioid system regulates numerous physiological functions, including responses to stress, and plays a key role in modulating mood and well-being, as well as addictive behaviours via reward processing [27].

Victor et al. [15] sought to examine whether self-harm is an “addiction” by comparing substance use and self-harm using one identified dimension of addiction, craving. Results found craving scores for substances occurred across a variety of contexts and were significantly higher than craving scores for self-harm. They concluded that self-harm appears to be craved only in the context of the removal of negative emotions and that findings advocate for an emotional regulation model of self-harm, as opposed to an addictive behaviour model. However, craving is only one of the diagnostic criteria for addictive behaviour within both the ICD-11 and DSM-IV. Others include impaired or loss of control, increasing priority given to behaviour, and continuation or escalation of behaviour despite negative consequences.

A recent review of the literature [19] concluded that self-harm can be conceptualised as an addiction and advocates for the use of an addictive model of self-harm. The review summarises research highlighting the role of neurobiological and psychological mechanisms in the development of an “addiction” to self-harm behaviours. The authors theorise that, when an individual engages in self-harm, opioid and dopaminergic systems are activated [17], providing brief relief from emotional and psychological pain.

Further support for repetitive self-harm as an addictive behaviour can be found within research evidencing the use of the opiate antagonist naltrexone hydrochloride as an effective pharmacological treatment of self-harm behaviours. Naltrexone is an opioid-receptor antagonist developed as a medication to primarily treat substance use disorder, initially heroin addiction, through a reduction in cravings and feelings of pleasure associated with using the substance. This drug has since been used to effectively treat repetitive self-harm [28] and supports hypotheses that the endogenous opioid system plays a maintaining role in repetitive self-harm. A quantitative synthesis of studies investigating the use of naltrexone as a treatment for self-harm behaviours found 80% of participants reported a reduction in SH behaviours with just under half of participants’ self-harm behaviours reducing by 50% or more [29]. However, there are a limited number of studies included in the evidence.

Given the limited number of empirical studies and conflicting results on the addictive properties of repetitive self-harm, it is important to understand what is maintaining engagement in self-harm. This study aims to explore what maintains repetitive engagement in self-harm, whether repetitive self-harm is experienced as an addictive behaviour and, if so, if it is maintained by similar processes to other addictive behaviours, including preoccupation, loss of control, urges and craving, tolerance, lying about behaviour, ritual and negative impacts.

## Materials and methods

Ethical approval was granted by the University of Hertfordshire Health, Science, Engineering and Technology Ethics Committee; Protocol number: aLMS/PGT/UH/04978 [2].

Constructivist grounded theory, as described by Charmaz [30], was used to explore whether repetitive self-harm is experienced as an addictive behaviour and maintained by similar processes to other addictions. Due to limited research exploring the addictive or reinforcing properties of repetitive self-harm, a qualitative method was used. Grounded theory has previously been used to explore self-harm within non-binary adults [31]. Grounded theory is a credible and rigorous method, concerned with the systematic development of theory through the inductive process of simultaneous data collection and analysis. Constructivist Grounded Theory prioritises abstract understanding and advocates rigorous reflexivity from the researcher through critique of methodological decision making. This methodology was selected as it assumes that processes are constructed and acknowledges the role of the researcher in the analysis of data. To address the research question and aims, consideration needed to be given to how, why, and in what contexts self-harm may be conceptualised as an addictive behaviour or within an addictive behaviour framework.

A critical realist epistemological stance was adopted throughout, which assumes that realities of social processes are mediated through lenses of language, experiences, and social environments [32]. Critical realism stipulates that it is impossible to investigate or critically analyse a concept from an ‘objective’ researcher position, as we are unable to separate our own perspectives and realities.

Extensive consideration was given to how the conceptualisation of repetitive self-harm as an addictive behaviour could be introduced and explored with participants in an unbiased way. The authors reflected on the power and influence researchers hold within an interview setting when introducing a concept and decided that, to allow participants to share differing experiences and their own conceptualisation of repetitive self-harm, it should not be introduced by the researcher. Instead vignette methodology was employed to introduce these concepts. Initially, the authors sought to create audio vignettes that presented self-harm as an addictive behaviour from publicly available media. Unfortunately, publicly available sources on self-harm mirrored the lack of consideration of self-harm as an addictive behaviour found in the literature and did not present or probe the current and most widely accepted definition of addictive behaviour found clinically and within research. Therefore, the authors decided to hold and record a focus group with people with lived experience of addiction, and that questions should be designed to elicit audio clips that could be edited into short audio vignettes, to be played to participants.

Due to large discrepancies within the literature of what constitutes an addictive behaviour, it was decided that the current world diagnostic standard criteria of addictive behaviours, as defined by ICD-11, would be used to guide the research. To create the audio vignettes, a recruitment poster was shared on social media and with a local service user research group, seeking individuals with lived experience of addiction. A list of questions was created based upon the diagnostic criteria within the ICD-11, which invited focus group members to discuss their experience of each criterion. In July 2022, a focus group was held with four individuals of varying ages (35–65 years): one male and three females. The focus group was audio and video recorded and lasted for approximately one hour. Audio clips were edited into succinct descriptions of focus group members experiences of addiction to create the vignettes. Audio vignettes varied in length from 30 seconds to one minute and 40 seconds. All eight vignettes were subsequently played to every participant in the same order.

Participant inclusion criteria were: individuals aged over 18 years; identifying as having experience of repetitive self-harm in their lifetime, and English-speaking. As the present study aimed to understand repetitive self-harm, the study team sought to specifically interview adults who had engaged in this behaviour over several years or on multiple occasions. Repetitive self-harm was defined as five or more times within a period of 12 months as informed by previous research conducted with non-clinical populations [1,5]. Remote participation afforded the opportunity for participants residing in countries other than the UK to participate; unfortunately, due to this being unfunded research, recruitment was limited to English-speaking participants.

A targeted recruitment strategy was employed, which included recruitment through relevant mental health charities, self-harm organisations, and social network sites who advertised the study. Recruitment began on 1^st^ of September 2022 and continued until 7^th^ of April 2023. Potential participants provided written informed consent and demographic information, and those eligible to participate were invited to a remote interview. Purposive sampling resulted in 15 participants.

### Interview process

The research team felt that vignette methodology would allow the lead researcher to present the ICD-11 diagnostic criteria of addictive behaviours impartially, invite participants to offer their own insights or hold a different opinion, and mitigate against acquiescence bias. There is a wealth of research highlighting the benefits of vignette methodology, particularly to allow the researcher to explore sensitive topics [33].

The use of a pilot interview with an Expert by Experience allowed for feedback on whether they felt the audio vignettes or follow up questions were priming or leading their responses in any way. The Expert by Experience fed back that they felt comfortable informing the researcher if their experiences were dissimilar to those shared within the vignette. The decision to create and use real life audio vignettes was taken as a way to focus the interview on the research aims and questions (whether repetitive self-harm is experienced as an addictive behaviour or not) in an open and non-leading way, where participants felt able to and were encouraged to disagree with material presented if it did not align with their experiences.

### Interview schedule

The most popular data collection method within qualitative research are semi-structured interviews, allowing the researcher to develop broad, open-ended questions that can begin to focus the interview, whilst allowing for flexibility to explore an individual’s experience of the process under investigation. A semi-structured interview schedule was used to ensure consistency of questions and presentation of material across the interviews. The flexible nature of the interview schedule compliments a Grounded Theory analysis of data as it allows the interviewer to complete the iterative process of data analysis alongside data collection, and the opportunity to amend the interview schedule in order to seek further information on tentative categories identified within previous interviews.

In line with Grounded Theory methodology, the interview schedule must not impose the researchers position or understanding of the topic upon participants from the outset. Instead, flexible questions allowed for the interview to explore the participant’s reality. After listening to each audio vignette, participants were asked to respond in their own time with their perspectives on how these fit, or not, with their experiences of repetitive self-harm. Open questions were used throughout to allow participants to explore their experiences and social processes related to repetitive self-harm. In line with the research aims, the schedule included questions around the individuals’ thoughts, feelings, and contexts, and explored the “what”, “when”, and “how” of their experiences. Follow up questions pertaining to a participant’s experience of repetitive self-harm as addictive were only asked if/when the participant had introduced this to the interview themselves.

Interviews recordings were made via Zoom and transcribed using the inbuilt function. These were checked for accuracy and amended as appropriate, including anonymising them by removing any potentially identifying information such as names prior to storage by the first author. Once interviews were transcribed, recordings were permanently deleted. Anonymised transcriptions were stored on a secure, GDPR Compliant, two-factor authenticated OneDrive in Microsoft Word documents for analysis. Participants provided informed consent for data to be used only in the described study and for its secure storage for a five-year period (until 22/9/28).

### Data analysis

NVivo 12 software was used to organise, manage, and analyse the data.

Reflexivity is concerned with an ongoing dialogue and increased awareness and ownership of the researcher’s positionality [34]. Due to the active role of the researcher in constructing theory [35], bracketing was used to acknowledge, record, and set aside existing beliefs and biases [36]. The first author is a white, 30-year-old final year trainee clinical psychologist, with lived experience of repetitive self-harm. Throughout, this position was considered as an ‘Insider-Outsider’ [37], and the role this played within analysis of the data and the construction of findings was considered. Reflexive techniques were employed, such as regular supervision with the principal supervisor (JH) to continually question whether any information was privileged or ignored due to alignment or disagreement with the first author’s own beliefs and experiences. A reflexive journal was also utilised to reflect on assumptions made and data that stood out.

The data was analysed using an iterative process of coding alongside ongoing data collection. Data was analysed using initial line by line coding [30] and tentative codes were created to encapsulate the processes described by participants [38]. Focused coding then grouped initial codes into conceptual categories, deciding which codes best account for the data and are most theoretically relevant to the research question and aims. Memo-writing was used throughout to identify initial codes that occurred more frequently, or those that presented a novel theoretical idea about repetitive self-harm, and to develop early comparisons. Analysis moved from focused codes into conceptual categories as guided by Charmaz [30].

As exploratory codes were constructed, theoretical sampling was employed to elaborate and refine initial theoretical categories. With the seven initial participants identifying as female, social media platforms were used to readvertise the study seeking participants who identified as male in an attempt to recruit a representative sample.

Diagramming was used to visually represent categories generated from the data and their relationships, allowing the direction of categories to be presented and considered in an active, fluid way. Theoretical coding was then employed to analyse relationships between constructed categories and subcategories.

Member checking is recommended as a method of ensuring rigor within qualitative research [39]. Therefore, once a tentative grounded theory model was developed, it was shared with all participants as part of the member-checking process. Two new participants were also recruited to complete model-checking interviews. Within model checking interviews, the Grounded Theory model and the proposed interactions between categories, was verbally discussed with new participants. Participants were asked to discuss whether they felt it made sense with their experience or not and whether they believed it was a good framework to supporting thinking about repetitive self-harm. This feedback, as well as the results of model-checking interviews, contributed to the further development of the model and indicated credibility of results as participants stated the model and constructed categories were in line with their experiences of repetitive self-harm.

## Results

**Fig 1:**
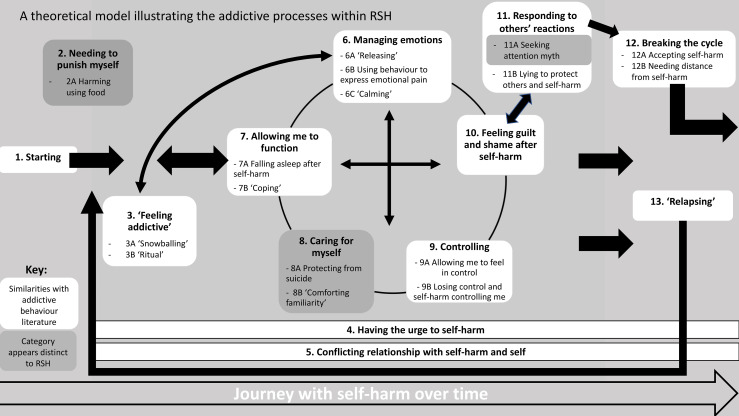
Theoretical model of addictive processes within Repetitive Self-Harm informed by Grounded Theory.

Participants’ demographics are presented in Table 1. A model was developed with data from 13 interviews and two model-checking interviews. Twelve categories were co-constructed, forming the overall model. Categories and subcategories are presented in Table 2; the overall model is presented in Figure 1 and outlines the interaction and relationships between each category.

**Table 1:** Participant demographic information.

Participant pseudonym	Age	Gender	Ethnicity	Received treatment from a physical or mental health setting for SH?	Currently engaging in RSH?	Diagnoses mentioned within interview
Philly	20	Female	British/German	Yes	No	History of eating disorder
Ceri	24	Female	White/English	Yes	Yes	Borderline Personality Disorder
Morgan	50	Female	White/Greek	Yes	Yes	Autism Spectrum Disorder
Leanne	44	Female	White/English	Yes	Yes	Attention Deficit Hyperactivity Disorder
Hannah	36	Female	White/English	No	Yes	Complex trauma
Alana	26	Female	White/English	Yes	No	History of eating disorder
Kate	36	Female	White/English	Yes	Yes	Autism Spectrum Disorder
Molly	25	Female	Black or Black British – African	Yes	No	None
Jake	29	Male	White/English	Yes	Yes	Depression
Beth	39	Female	White/English	Yes	No	None
Dan	61	Male	White/English	Yes	Yes	Depression
Chaya	29	Female	Ashkenazi (Jewish)	Yes	No	Depression
Sandy	25	Female	Malay	Yes	Yes	None
May	24	Female	White/English	Yes	No	None
Ellie	29	Female	White/English	No	No	None

**Table 2:** Categories and subcategories.

Category	Subcategories	Participant quotes
**1. Starting**		*“I started and it kind of escalated”* *“It first started because I got really angry”*
**2. Needing to punish myself**	2A Harming using food	*“I would just go back and pick at it, it was like I was punishing myself for how I was feeling, and the things that I was doing and so it didn’t feel right that it was healing. So, I would continually try to make it worse”* *“I viewed it like atoning for my sins, it was like anytime I thought I was a bad friend or a bad person I would be like well that doesn’t matter because you’ve punished yourself for it”* *“I tend to try and starve myself at that point, it’s kind of like I want to hurt myself in as many different ways as possible”* *“I am a smart girl, I have found ways you know, if I don’t have access to a cutter, I have found the vomiting”*
**3. ‘Feeling addictive’**	3A ‘Snowballing’	*“At some point I became really aware that… I was quite addicted to it. Yeah, if I gave into it too much, it was definitely less effective and even was becoming a full-blown addiction. It was all the kind of standard stuff, needing it more frequently, needing it to just function…really unexpected kind of physical withdrawal symptoms from it.”* “*Initially when you choose to first do it, there is definitely a reason for it and you make that decision, but then a few months into it, it definitely became something I did sometimes out of boredom or because I felt like I had to and that definitely feeds into the unconscious idea of it”*
	3B ‘Ritual’	*“a ritual. You have a particular way that you do it. You have a particular time that you do it and you have a particular place that you do it. People don’t do it sporadically; they have a particular safe place for them to do it”* *“It does feel sort of grand sometimes, but I wouldn’t think of that as a ritual. If I think of a ritual, it is something special so I guess that is how I would see it, but not in a ritual way in that I do it in a certain way in a specific time”*
**5. Having the urge to self-harm**		*“It’s been 7 years now, just over 7 years, with non-stop twenty-four-seven self-harm urges that their absence is impossible to imagine. When I try to explain to professionals about what the urges feel like, quite often you just see the horror on their faces. You know, it is so powerful, and it’s got such a strong pull on you”*
**6. Conflicting relationship with self-harm and self**		“*I viewed it as an enemy definitely when I was in it because I was so angry at myself for doing it. But even now, I do think it is something that I know so intimately, and it knows me so intimately, I have a relationship with it that I don’t have with anyone that I know in person, and no one will know of that relationship either, which I think keeps it being such an intimate thing”*
**7. Managing emotions**	6A ‘Releasing’	*“It’s kind of like your emotions are getting bigger and bigger inside, and you’ve just got to hit yourself to release the pressure almost building up inside you”*
	6B Using behaviour to express emotional pain	*“The reason I continued and the phrase that always went through my mind was that it was an outward expression of inward pain”*
	6C ‘Calming’	*“That feeling of relief and clearness and clarity, and like relaxation, and it literally would feel like, if I could describe it going from like burning hot to this nice, cool feeling throughout my body. Just relaxed and not worried”*
**10. Allowing me to function**	7A Falling asleep after self-harm	*“Why do I do it? The first answer that came up is to help me sleep, you know, the racing thoughts making actually getting to sleep quite difficult”*
	7B ‘Coping’	*“It is a massive coping mechanism, more effective than anything else I could have done in that moment. For me, the calm that came after was that like, okay, now you get on, now you move.*
**12. Caring for myself**	8A Protecting from suicide	*“Self-harm keeps me from killing myself. You know I’m doing what I need at this point, because I know the alternative is actually far worse than this. So, I need to stop myself from getting there, I need to protect myself from trying to kill myself”*
	8B Comforting familiarity	*“Like a crutch from back in the day, it’s like an old familiar thing, I guess. Like pulling out your favourite blanket or something like that, it’s the old familiar”*
**13. Controlling**	9A Allowing me to feel in control	*“I could control what I did, and I could control how to fix it. Because everything else was out of control and fake. My mind always felt and does always feel like it is out of control, it was something that gave me more control”*
	9B Losing control and self-harm controlling me	*“Before I did it for the first time I was in control because I chose not to. But from the first time I did it, I was not in control anymore. To be honest, I think I knew I wasn’t in control, but I wanted to feel like I was in control, and I wanted to take control over it. But basically, everything I did in order to feel like I had control of it just showed that I had no control”*
**15. Feeling guilt and shame after self-harm**		*“Ironically, sometimes for doing it you would be like oh I was really stupid! I shouldn’t have done that. So now I am going to do it some more to tell myself off for doing it”*
**16. Responding to others’ reactions**	11A Seeking attention myth	*“I can tell you what it isn’t. It isn’t acting out. When I was younger there was a lot of, I still hear it now, are you doing it for attention? I would be absolutely mortified if people found out so that is not why I’m doing it.”*
	11B Lying to protect others and self-harm	*“Function of lying was also to keep it protected it was mine! It wasn’t to be shared with other people. It was about me, and it was something that I had, and I didn’t want to invite other people’s commentary or opinions on it. So, I used it to protect the self-harm as well”*
**18. Breaking the cycle**	12A Accepting self-harm	*“I suppose it’s kind of like admitting its self-harm is the first stage of trying to get over it, like admitting you’ve got a problem, like that stage”*
	12B Needing distance from self-harm	*“You don’t realise how bad it is until after. And when I say after I don’t mean like a couple of months. I mean like for me obviously it has been nearly 10 years now and I can look back and think God! That was actually really significant as an experience”*
**19. Relapsing**		*“I only ever had one relapse, and it was like a singular relapse. It wasn’t kind of like I relapsed for three months and then stopped again. I had a singular incident, and then it’s never happened since”*

### The model

Through Grounded Theory analysis, a tentative theoretical rendering of the data was developed and is presented as a model in Fig 1. Participants described the processes that kept them engaging in repetitive self-harm. The interconnecting arrows are used within the model to represent that participants’ journeys with repetitive self-harm were not linear, instead processes that maintain engagement were reciprocal, reinforcing of one another, and fluid over time.

Participants described their experiences of starting self-harm; for many, feeling a ‘need to punish’ themselves either led to starting or occurred soon after. Participants quickly felt they were no longer always consciously choosing to engage in repetitive self-harm and began feeling it was ‘addictive’. Once participants had engaged with self-harm, they continued to have urges even after stopping for many years and experienced a conflicting relationship with repetitive self-harm. They described that both the urge to self-harm and conflicting relationship with repetitive self-harm did not end once they stopped engaging in this behaviour. Instead, they described feeling this would be something they would always experience, long after stopping.

Throughout each incidence of self-harm, participants experienced processes that interacted, describing this as the “cycle of self-harm”. Self-harm was used to manage feelings of overwhelming emotion and produced calming effects. This experience enabled participants to function, sleep, and feel they could cope with life. Over time, the process of harming oneself, the relief experienced immediately after, and care given to treating wounds began to be interpreted as their way of caring for themselves. It was described as comforting, familiar and, for some participants, protected them from ending their life because it allowed them to cope with distress and suicidal thoughts or ideation. At times, repetitive self-harm afforded a sense of control of the uncontrollable (emotions and external events) but at other times it became increasingly apparent to participants that they were no longer in control of their self-harm, instead experiencing it as controlling them. Effects of self-harm, such as releasing emotions or calming, were short lived and, quickly, participants experienced guilt and shame due to self-harm. For some, this reinforced feelings of needing to punish themselves and led them to harm again. Participants described these five processes as cyclical and interacting, which is why they are displayed within their own distinct cycle within the model and double headed arrows are used to show the interactions between each category.

Finally, participants detailed how others’ responses to their repetitive self-harm could either contribute to feelings of guilt and shame or support them to “break the cycle” of self-harm. They identified ways in which they found they could “break the cycle” of repetitive self-harm but also described experiences of “relapse”, which reinforced the cycle and were often experienced as more out of control than previous incidences of repetitive self-harm. The model displays which categories were similar to theoretical and empirical understandings of addictive behaviours (highlighted in white) and which appeared to be distinct to repetitive self-harm (highlighted in grey).

#### Category 1: Starting.

Participants described how quickly self-harm “*escalated*” after starting. They appeared to be describing a feeling that once one starts and experiences relief, there is no going back. One described, “*the first time is so crucial… it felt so taboo, but the second you cross the line of doing it the first time and you realise it is not as bad as you thought it was going to be, then it just becomes easier after that*” (May). They explained a feeling that once you have started: “*You can’t go back to how life was before; it is always there as an option, and I can’t undo that it has happened, and I can’t undo how it made me feel at the time*” (Alana).

#### Category 2: Needing to punish myself.

A need for or feeling that they are deserving of punishment characterised the motivation to start and continue harming for 11 participants. This was described as relating to low self-esteem, self-worth, or feelings of intense anger with themselves or others, which was then directed towards themselves. These feelings appeared to precede starting self-harm and contributed to the motivation to initially engage in self-harm. However, some participants observed and described a progression or increase in this once they had started.

One participant described this experience and how it was observed by others: “*I got to a certain age and this switch went on inside my head and from that point I was just hell bent on punishing myself. It almost did feel like a switch of this all-consuming rage with myself at times, and the only way to get it out was to feel that pain and to punish myself”* (Beth).

#### Category 3: Feeling addictive.

Twelve of the 15 participants described the addictive effects or feeling addicted to repetitive self-harm. Participants appeared to experience the feeling that, once you have started engaging in self-harm, “*you don’t want to stop. You just don’t want to*.” (Dan). They explained this experience using language typically used to describe addiction to substances, such as “*craving*”, and five participants described needing to increase their self-harm through harming more severely or frequently to experience the same effects. It appeared that participants were suggesting feeling addicted to repetitive self-harm began quite soon after first engaging in self-harm, continued to grow over time, and increased through repeated engagement. A feeling of being addicted to repetitive self-harm felt particularly relevant for participants who had been engaging in it for: “*well over 30 years, I started when I was a really young kid, so you don’t continuously do such a self-destructive behaviour if there isn’t some level of addiction there*” (Leanne).

#### Category 4: Having the urge to self-harm.

All participants described ongoing urges or thoughts to self-harm, whether they were still actively engaging with repetitive self-harm or had not self-harmed in many years. Continuing urges are depicted within the model using a solid line from starting self-harm and continuing even once an individual has broken the cycle. Participants reported these urges do not always present in the context of negative emotions and can occur even when things in their life feel okay. This experience was described as though the urges were intrusive thoughts: “*Even when I am otherwise feeling fine and dandy and strong, and I can deal with this, and the trigger isn’t even that severe. The thought is a bit intrusive. It comes up even when it’s not needed, you feel a magnetic pull, and you either can or you can’t stop yourself*” (Jake).

#### Category 5: Conflicting relationship with self-harm and the self.

Descriptions of participants’ experience with repetitive self-harm appeared to highlight a relational element where participants were speaking about self-harm as if it was another person. There was conflict within this relationship; at times repetitive self-harm was experienced as a supportive friend and at other times it felt more like an enemy who was hurting them. The relational element was described as “*toxic*” by some participants as the continual urges telling them to hurt themselves, or that they deserved to be punished, felt abusive. It is important to note that participants described these conflicting themes as coexisting, they described repetitive self-harm as both an enemy and a friend, it was not either or. This was also described as an ongoing relationship that does not end when you stop harming and is therefore depicted in the same way, using a solid line.

One participant captured this changeable relationship as they described feeling: “*It is a massive conflict. But I suppose that’s because it is something you feel like you shouldn’t do and then saying it feels really good, and it helps me to manage my emotions like what? But it does, it felt really good*” (Ellie).

#### Categories 6–10: The repetitive self-harm “cycle”.

All participants spoke in some way about the cyclical process that occurs during every incidence of self-harm. This was discussed during early interviews and an aspect of theoretical coding was to explore participants experience of this cycle. Categories are numbered for ease of interpretation; however, this was an interactive process with each category contributing to one another.

#### Category 6: Managing emotions.

All participants described overwhelming emotions or emotional pain as the antecedent to self-harm. One emotion described as particularly difficult to manage was anger, described by seven participants as a trigger due to feeling like they had no other way to control or stop this feeling. Repetitive self-harm became an effective strategy to manage the build-up of intense emotions and allowed participants to feel in control of their emotions: “*After trying all these different things and nothing was working, I worked out that if I self-harm at the right time, at the right point in the build-up, I can stop it going too far*” (Morgan).

#### Category 7: Allowing me to ‘function’.

Through repeated use of self-harm, and the ability it provided to manage emotions, participants began to feel that it allowed them to function and continue to engage well in other areas of their lives. Participants described quickly feeling reliant on repetitive self-harm to survive and manage life, repetitive self-harm allowed them to continue to present as ‘normal’ and be productive: “*I can function again. I often can’t function if I’m not self-harming. Self-harm often helps me to function*” (Morgan).

#### Category 8: Caring for myself.

Repetitive self-harm became participants’ way of taking care of themselves. They described prioritising this behaviour during times when they did not feel able to engage in basic acts of self-care, such as personal hygiene. This experience relates to other categories within the self-harm cycle as participants explained that as repetitive self-harm allowed them to manage their emotions, to function and stop them from feeling worse, then it is a way of caring for oneself. Similarly, to Category 5, participants spoke of being aware that they were harming themselves but feeling conflicted as it also became their way of caring for themselves: “*This is where you get the self-harm ritual and the self-care side of stuff start overlapping… because I see that hour as ‘me’ time. It’s for me, for nobody else and that’s part of the process… it gets to the point where this is almost self-care*” (Morgan).

#### Category 9: Controlling.

This category illustrates the polarising nature of repetitive self-harm, which participants described afforded them a sense of control but, in other ways, they felt as though they had lost control of their self-harm; instead, it was controlling them. It appeared feeling in control of self-harm was something that occurred soon after participants started. However, over time, they detected self-harm may no longer be under their control by noticing feeling that they needed to self-harm or taking risks with their repetitive self-harm (risking being caught or using more violent and dangerous methods). It is important to note that sense of control over repetitive self-harm was fluid and changing, it did not appear to be something you had and then lost. Instead, there would be periods where one had more control of it and when they did not. One participant described a point when they realised they were not in control: “*I was going to CAMHS [Child and Adolescent Mental Health Services] and my mum said please, why can’t you just stop? I had this moment in my head where I was like I can’t! But I’ve never considered that I couldn’t at all. It had always felt like a choice until it didn’t*” (Alana).

#### Category 10: Feeling guilt and shame after self-harm.

Following an incident of self-harm, after the initial feelings of release and calm, participants described that they quickly experienced intense feelings of guilt or shame. Feelings of guilt and shame after self-harm could contribute to needing to punish themselves (category 2), causing participants to feel worse and could start the process of building negative emotions again. Participants described that guilt and shame can occur within minutes after harming and continue to build due to the constant and visual reminder of harm. One participant described how feelings of shame reinforced their self-harm as it: “*Would pile on to everyday crap I was feeling and that would then trigger the next episode and the next. So as much as it would help me, it was also bringing on so many feelings of shame and guilt*” (Alana).

#### Category 11: Responding to others’ reactions.

All participants spoke of experiences of responding to others’ reactions to self-harm, including professionals. Ten participants described feeling stigmatised due to self-harm and reported that no one understood. Fear of judgement and stigma was given as one of the main reasons for not telling others or seeking help for repetitive self-harm. A number of participants had not previously told anyone about their self-harm but discussed examples of when strangers had commented on scars or asked about them. Participants described how others’ reactions, including professionals’ responses and use of language when they had sought help, were fundamental in either supporting them to reduce or stop repetitive self-harm or, at times, reinforcing and maintaining the behaviour. An example of this was use of the medical term “*superficial*” to describe self-harm injuries: “*When you’ve had someone say they’re superficial to you before, it does make you think that you’re almost doing it like wrong, like you’re not bad enough. You are not doing it deep enough*” (Ceri).

#### Category 12: Breaking the cycle.

Eleven participants had stopped repetitive self-harm at some point throughout their journey and then returned to self-harm after a period of abstaining. Five participants described periods where they tried to reduce or limit their self-harm. Six participants described reaching a point where they had had enough, or could no longer engage in this behaviour, and termed this ‘breaking the cycle’ of repetitive self-harm. One participant reflected upon what supported or enabled them to do so: “*The thing that actually broke the self-harm cycle was that I went on holiday, and I couldn’t do it because I was going to be with my grandparents. That broke it and then it was more sporadic, and I felt like I could control it from that point*” (Ceri).

#### Category 13: ‘Relapsing’.

Six participants spoke of their experiences of “relapsing” and returning to repetitive self-harm after not engaging for some time. They described a worry or anxiety that, because repetitive self-harm was how they coped in the past, they would return to this behaviour in future; the experience of a sliding scale that would inevitably result in self-harm. For one participant this was experienced as: “*There is always that fear that… no matter how long it’s been since my last really bad episode, in my head it could just go, and if it does then it will come back*” (Leanne).

## Discussion

This study is the first to utilise constructivist grounded theory to explore the addictive processes within repetitive self-harm. Many people conceptualised their repetitive self-harm as addictive and described having ‘urges’ and ‘relapses’. Repetitive self-harm started out as a choice for many but then became something that they could not stop. Repetitive self-harm seemed to coexist with both a positive function, helping people to manage their emotions and as a form of self-care, and a negative function, feeling controlled by repetitive self-harm, guilt, and shame.

The Rational Informed Stable Choice model of addiction [40] suggests that initial choice to engage in a potentially harmful behaviour always involves consideration and evaluation of the options. Similarly, the current findings evidence that individuals start self-harm with an expectation or hope of potential benefits and an acceptance of adverse consequences [41]. The predominant focus of self-harm research is around how to treat this behaviour. However, results of this study suggest that more consideration should be given to reasons why individuals start harming alongside focus on reducing harm initiation. Further research exploring the decision-making process when first engaging in self-harm, possibly using a mixed sample of individuals with experience of self-harm and those without, would be valuable.

Results of this study align with the Defective Self Model of NSSI [42], which proposes that individuals engage in repetitive self-harm due to feelings of low self-worth and the belief that they are in some way deserving of harm. Additionally, the results provide support for the self-punishment model of self-harm [43] and findings from previous research around motivations for self-harm [43,44]

Results from this study suggest that individuals currently engaging in repetitive self-harm experience intense or consistent urges, whereas those who had stopped for some time described fleeting thoughts or urges to reengage. This could be understood within the Cognitive Model of Drug Urges [45]. The current study builds upon self-harm literature evidencing that urges or cravings commonly experienced by those who self-harm increase with age, that intense urges predict more frequent self-harm behaviour [46], and self-harm is experienced as more reinforcing through repeated exposure [47].

Similarities can be seen between participants’ descriptions of needing to self-harm more frequently, or engaging in more risky harm behaviours, to the well-evidenced concept of tolerance. Tolerance is defined within the DSM-5 [15] and ICD-11 [2] as the need for greater amounts of substance to achieve the desired effect. Whilst Victor et al. [48] concluded that self-harm appears to only be craved in the context of the removal of negative emotions and findings advocate for an emotional regulation model of self-harm rather than an addictive model, this study concludes that urges to self-harm were experienced within a number of different contexts and advocates for a more complex and nuanced understanding. The differences in findings may have occurred as Victor et al.’s [48] research was limited to only one identified dimension of addiction, craving.

A conflicting relationship between the individual and repetitive self-harm was pervasive across many categories within the model. Each element or function of repetitive self-harm had a reciprocal role: as friend and enemy, it makes me feel better but it also makes me feel worse, it is comforting but it hurts. This finding aligns with previous research into excessive alcohol use [49] and relational models of addiction [50]. It is thought that efficacy of peer-led recovery interventions, such as Alcoholics Anonymous, is largely due to the relational components offered, providing a sense of belonging and a reduction in guilt and shame [51]. Participants highlighted the potential benefits of third sector and lived experience led organisations, such as Battle Scars, and benefits of peer support, such as gaining an understanding of self-harm and support from others with similar experiences [52].

The results of this study also support previous findings around self-harm becoming an act of self-care [53]. This conceptualisation could shift the current narrow characterisations of self-harm as an impulsive act of violence towards oneself [54]. This study also found that descriptions of repetitive self-harm as “*superficial*” by professionals contributed to participants feeling they were not harming severely enough and possibly led to escalation of these behaviours. Clinically, particular attention should be given to language used to describe repetitive self-harm behaviours.

The finding that individuals experience a loss of control over their repetitive self-harm behaviours overlaps with the ICD-11 diagnostic criteria for addiction of a loss or impairment of control over the behaviour (e.g., onset, frequency, intensity, duration, termination, context). Results support dual process models evidencing the role of both automatic (unconscious) and controlled (conscious) processes within repetitive self-harm, which have previously been evidenced in addiction [55].

Category 11 (responding to other’s reactions) evidences how social reactions to repetitive self-harm behaviours are significant in the process of stopping, previously suggested by Orford [56]. Treatment models of addiction, such as the stages of change model [57], may be applied to repetitive self-harm to provide a framework for understanding change. Similarly, motivational interviewing has been evidenced as an effective treatment within addiction and addictive behaviour [58] and may also be useful when applied non-judgementally within the treatment of repetitive self-harm. Further research is required to explore the efficacy of this as an intervention for repetitive self-harm. Training for all supporting those who repetitively self-harm, and specifically mental health teams, to address the current biases and stigma relating to self-harm could be beneficial. Viewing self-harm through an addictive behaviour lens could address some of the narrow definitions of what constitutes self-harm, for example the current gendering of self-harm. Further research around the role of culture and ethnicity within repetitive self-harm behaviours and help seeking is also required to inform appropriate interventions.

Within clinical practice, the grounded theory model developed here could be used as a model of repetitive self-harm to guide discussions during assessment and formulation stages of treatment. In particular, working alongside the client to identify what stage they are at in relation to changing their self-harm behaviours or incorporating “relapse” into clinical safety planning when an individual wishes to stop.

It is important to note the strengths and limitations of this study. A strength of this research was the inclusion of participants who were currently engaging in repetitive self-harm, supporting the clinical utility of findings and implications. A further strength was the use of realistic and believable vignette scenarios [33], developed from a focus group with individuals with lived experience of addictive behaviours. Participants were of a variety of ages, which could mean that findings can be applied to clinical work with adults who engage in repetitive self-harm across the lifespan. Finally, when asked about their experiences participating in the research, many participants reported they found it to be validating and interesting.

Whilst the sample size was impressive for a grounded theory study, the results are based on a sample of adult participants with limited cultural and ethnic diversity, thus limiting the transferability of findings [59]. The use of a self-selecting sample (with all but one residing in the UK) may limit the transferability of findings beyond a Westernised view of repetitive self-harm. The sample included only two male participants; therefore, findings may not be representative of the male experience. Future research should strive to mitigate against the gender bias within self-harm literature and assess the replicability of current findings within an all-male sample.

Results of this study highlighted the potential long-term benefits of increased funding and training for primary care services and therapies available to those who self-harm. This could result in significant long-term cost-saving to the NHS in lieu of the current presentation and costs in the form of surgical treatment of self-harm [60], 24-hour crisis team support, or hospitalisation of those who engage in repetitive self-harm [61]. Early intervention and therapeutic support for those who self-harm is in line with the national suicide prevention strategy for England.

Results of this study invite policymakers to consider funding community, lived experience led projects and organisations who are already providing services for those who engage in repetitive self-harm. Increased funding to these organisations is paramount as charitable organisations are reliant on funding that has been decreasing exponentially during years of austerity policies [62,63]. In addition, services providing mental health support for self-harm appear incredibly reluctant to offer peer support groups potentially as a result of concerns around the ‘social contagion’, also referred to as ‘suicide contagion’ of repetitive self-harm [64,65].

The ‘social contagion’ model of SH suggests that by witnessing both online and real-life discussions or portrayals of self-harm, others (particularly adolescents) are more likely to ‘copy’ or engage in this behaviour [66,67]. Unfortunately, these understandable and valid concerns around online safety may inadvertently contribute to the stigma and shame described by participants within this study, thus reinforcing the repetitive self-harm cycle. These narratives may also overestimate potential harm and underestimate possible benefits of peer support, such as gaining an understanding of their self-harm and support from others with similar experiences [68].

Results of this study support literature that posits alternative models for the consideration of self-harm content [68], as participants identified that acceptance and finding alternatives supported them to break the repetitive self-harm cycle. One must also consider the impact of using evocative language, such as ‘social contagion’ or the framing of self-harm content as ‘causal’, when discussing a population shown to already experience low self-worth [69] and high levels of guilt and shame [70]. This language continues to locate blame for self-harm in the individuals engaging in the behaviour, whilst often ignoring the wider social and political contexts all forms of media reflect. Findings of this study suggest that broad governmental statements or policies for social media platforms to limit or remove all self-harm content are at risk of causing unintentional harm. Further research is required exploring the benefits of peer support (both online and face-to-face) to inform future clinical treatment options, policy, and guidance.

This study has provided a conceptual model of the processes that maintain engagement in repetitive self-harm and illustrates similarities and overlap between repetitive self-harm and addictive behaviours. This work provides evidence for the potential benefits of conceptualising repetitive self-harm as an addictive behaviour, in particular drawing upon the wealth of models to understand, treat, and recover. The current study highlights a need for increased understanding of the addictive processes within repetitive self-harm.
